# The roles, mechanisms, and therapeutic implications of glucocorticoids in glucose and lipid metabolism

**DOI:** 10.1016/j.mmr.2026.100037

**Published:** 2026-05-15

**Authors:** Qiu-Yu Xu, Jie Zhang, Xin-Jian Wan, Ling Wu, Ji-Chun Yang, Yan Lu

**Affiliations:** a Institute of Metabolism and Regenerative Medicine, Digestive Endoscopic Center, Shanghai Sixth People’s Hospital Affiliated to Shanghai Jiao Tong University School of Medicine, Shanghai 200233, China; b Department of Assisted Reproduction, Shanghai Ninth People’s Hospital, Shanghai Jiao Tong University School of Medicine, Shanghai 200011, China; c Department of Physiology and Pathophysiology, School of Basic Medical Sciences, State Key Laboratory of Vascular Homeostasis and Remodeling, Center for Non-coding RNA Medicine, Peking University Health Science Center, Beijing 100191, China

**Keywords:** Glucocorticoids (GCs), Glucocorticoid receptor (GR), 11β-hydroxysteroid dehydrogenase type 1 (11β-HSD1), Metabolic diseases

## Abstract

Glucocorticoids (GCs), steroid hormones synthesized and secreted by the adrenal cortex, have been the subject of intensive scientific investigation for over eight decades due to their critical roles in physiological and pathological processes. The systemic secretion, bioavailability, and tissue-specific concentrations of GCs are strictly orchestrated by the hypothalamic-pituitary-adrenal axis, serum corticosteroid-binding globulin levels, and the enzymatic activation of 11β-hydroxysteroid dehydrogenase type 1 and 2 (11β-HSD1 and 11β-HSD2). As pivotal metabolic messengers, GCs signal through binding to glucocorticoid receptors (GR) to maintain metabolic homeostasis by modulating the transcriptional landscape of genes central to glucose and lipid metabolism. Any imbalance in GC levels, whether excess or deficiency, leads to the development of diverse metabolic disorders. Elucidating the underlying mechanisms of this metabolic regulation remains a primary objective of contemporary research. This review summarizes landmark discoveries and focuses on the metabolic impact and dysfunction of glucocorticoids, aiming to establish a robust foundation for the development of optimized therapeutic interventions for patients with metabolic diseases.

## 1 Background

**Fig. 1:**
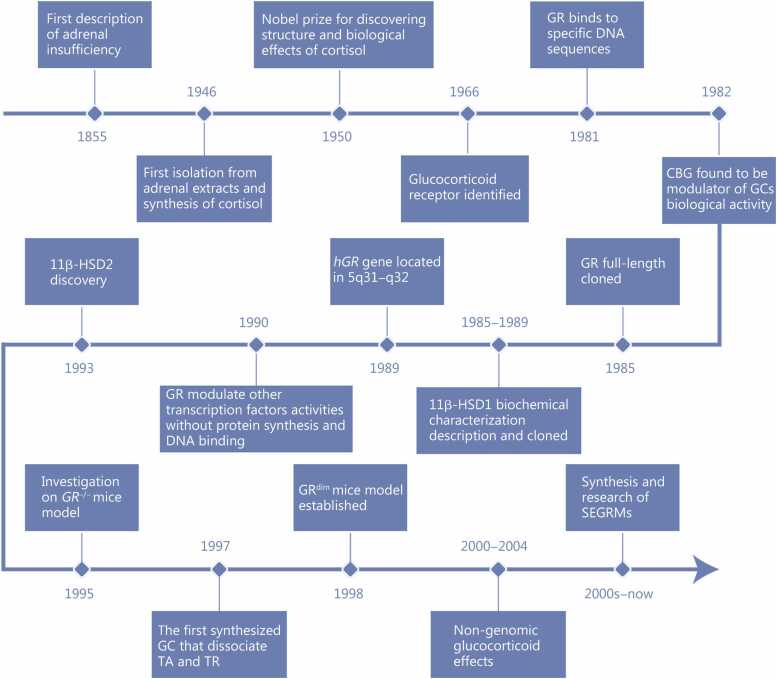
Timeline of GC discoveries. The node “2000–2004” denotes the period during which research on non-genomic glucocorticoid effects entered a systematic phase, characterized by mechanistic studies of rapid, transcription-independent GC actions. The node “2000s–now” represents the concurrent but distinct research trajectory focused on the synthesis and development of SEGRMs. The temporal overlap reflects the parallel emergence of these two independent lines of investigation rather than redundancy. GC. Glucocorticoid; GR. Glucocorticoid receptor; 11β-HSD. 11β-hydroxysteroid dehydrogenase; SEGRMs. Selective glucocorticoid receptor modulators; *GR*^-/-^. *GR* knockout; GR^dim^. GR Dimerization-deficient; TA. Transactivation; TR. Transrepression; CBG. Corticosteroid-binding globulin.

Research into the glucocorticoids (GCs) commenced with the first description of adrenal insufficiency in 1855 by Thomas Addison [1]. A major milestone in GC research came in 1946 when Edward C. Kendall isolated five steroid compounds from adrenal extracts, including Compound E, subsequently identified as cortisol, the predominant GC in humans, and Lewis H. Sarett successfully synthesized it chemically [2]. The field gained international recognition in 1950 when Kendall, Tadeus Reichstein, and Philip S. Hench received the Nobel Prize in Physiology or Medicine for their discoveries on cortisol structure and biological effects [3]. The following decades witnessed critical advances, including the identification of specific glucocorticoid receptors (GRs) in 1966, the demonstration of GR binding to DNA in 1981, the discovery of corticosteroid-binding globulin (CBG) in 1982, and the cloning of GR and 11β-hydroxysteroid dehydrogenase enzymes (11β-HSD1 and 11β-HSD2) in the late 1980s−1990s [4]. The development of *GR* knockout (*GR*^⁻/⁻^) and GR dimerization-deficient (GR^dim^) mouse models enabled functional studies of GR-mediated genomic and non-genomic signaling pathways in 1995 and 1998, respectively, while the 2000s brought recognition of nongenomic GC actions and selective GR modulators [5], [6]. The timeline of these pivotal discoveries is illustrated in Fig. 1.

## 2 Glucocorticoid biosynthesis, regulation, and bioavailability

GCs are steroid hormones synthesized and secreted by the zona fasciculata of the adrenal cortex, a process stringently governed by the hypothalamic-pituitary-adrenal (HPA) axis [7]. The hypothalamus secretes corticotropin-releasing hormone (CRH), which stimulates the pituitary gland to release adrenocorticotropic hormone (ACTH). ACTH then binds to melanocortin-2 receptors (MC2R) on adrenal cortical cells, triggering GC production [8]. GCs exert negative feedback on the HPA axis by suppressing CRH and ACTH expression [9]. Additionally, extra-adrenal GC synthesis occurs in peripheral tissues (e.g., thymus, intestine, and skin), where it orchestrates tissue-specific physiological effects [10]. Once secreted into the circulation, GCs act as essential regulators of mammalian physiology, controlling critical processes including growth and development, metabolic homeostasis, immunomodulation, water-electrolyte balance, and reproduction [11].

**Fig. 2:**
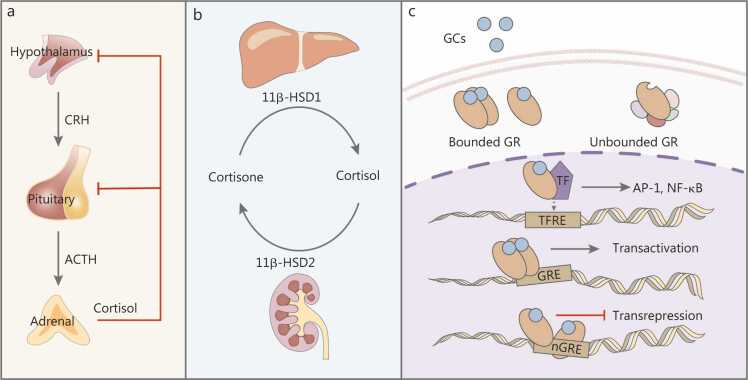
Modification of GC secretion bioactivity and function. **a** GC secretion modulated by the HPA axis. The hypothalamus releases CRH, which stimulates ACTH secretion from the pituitary, subsequently inducing cortisol production from the adrenal gland. Elevated cortisol exerts negative feedback on both the hypothalamus and pituitary to suppress further GC release. **b** Peripheral regulation of GC bioactivity by 11β-hydroxysteroid dehydrogenases (11β-HSD). 11β-HSD1, predominantly expressed in the liver, converts inactive cortisone into active cortisol, thereby amplifying local GC signaling. Conversely, 11β-HSD2, expressed mainly in the kidneys, inactivates cortisol back to cortisone, protecting mineralocorticoid-sensitive tissues from GC excess. **c** GC genomic effects. GCs diffuse into cells and bind cytosolic GRs (bound GR), while unliganded GRs remain in an inactive state (unbound GR). Ligand-bound GRs translocate into the nucleus and regulate gene expression via three mechanisms: i) monomeric GR interacts with transcription factors (TF) such as AP-1 and NF-κB at transcription factor response elements (TFRE), indirectly repressing proinflammatory gene expression; ii) dimerized GR binds glucocorticoid response elements (GRE) to transactivate anti-inflammatory target genes directly; and iii) GR binds negative glucocorticoid response elements (nGRE) to repress gene transcription directly. CRH. Corticotropin-releasing hormone; ACTH. Adrenocorticotropic hormone; GC. Glucocorticoid; GR. Glucocorticoid receptor; AP-1. Activator protein-1; NF-κB. Nuclear factor κB; nGRE. Negative glucocorticoid-response element.

During homeostasis, GC release is controlled by circadian and ultradian rhythms, with peak GC levels typically occurring in the early morning [12] (Fig. 2**a**). Once released into circulation, less than 5% of GCs remain unbound; more than 80%–90% of circulating GC (predominantly cortisol in humans) is bound to CBG, while albumin binds to less than 10%–15% of the remaining GC with low affinity [13]. Based on the free hormone hypothesis, only unbound GCs are biologically active, as they can readily bind to GRs in target cells. Therefore, CBG levels critically determine GCs’ bioavailability [14].

Beyond CBG-mediated regulation, local tissue GC bioavailability is further modulated by two isoforms of 11β-HSD. 11β-HSD1 is a bidirectional enzyme that predominantly converts inactive cortisone to active cortisol, whereas 11β-HSD2 catalyzes the irreversible conversion of cortisol to cortisone [15]. GCs have approximately 10-fold greater affinity for mineralocorticoid receptors (MRs) than for GRs. Consequently, under basal conditions, low-dose GCs preferentially bind MRs rather than GRs [16]. To prevent inappropriate GC-mediated MR activation in tissues with high MR expression (e.g., kidney), these tissues express elevated levels of 11β-HSD2, which locally inactivates GCs and thereby preserves MR specificity for aldosterone signaling. Conversely, 11β-HSD1 is predominantly expressed in the liver, adipose tissue, and skeletal muscle, where its dominant reductase activity amplifies cortisol (the primary glucocorticoid in humans) signaling [17]. Through the differential effects of these two isoforms, GCs are precisely regulated in a tissue- and organ-specific manner (Fig. 2**b**).

In addition to the intrinsic regulatory mechanisms governing GC bioavailability, HPA axis activity is also shaped by a broad range of extrinsic factors. Physical stressors that activate the HPA axis include fasting, trauma, major surgery, sleep deprivation, and sepsis [18]. Psychological stressors, such as chronic stress, adverse childhood experiences, and psychiatric disorders (e.g., depression and post-traumatic stress disorder), lead to HPA axis dysregulation [19]. Environmental factors, including noise and pollutants, also impact normal HPA axis function [20], [21].

## 3 Mechanisms of glucocorticoid action

GCs elicit their biological effects by binding to GRs, which are encoded by nuclear receptor subfamily 3, group C, member 1 (NR3C1) and exist in multiple isoforms, of which GRα is the canonical receptor mediating GC-induced transcriptional regulation, while GRβ acts as a dominant-negative inhibitor contributing to GC resistance [22]. The GR comprises three functional domains: the N-terminal transactivation domain (NTD), the DNA-binding domain (DBD), and the ligand-binding domain (LBD), and resides in the cytoplasm as part of a multiprotein complex with heat shock proteins (HSP70 and HSP90) and other molecular chaperones [23]. Upon ligand binding to the LBD, GR undergoes conformational change, translocates to the nucleus, and interacts with glucocorticoid response elements (GREs) or negative GREs (nGREs) of target genes via the DBD, thereby mediating transcriptional activation or repression (Fig. 2**c**) [24]. GR can also regulate gene expression through a “crosstalk” mechanism that involves interactions with other transcription factors rather than direct DNA binding [25]. *GR*^⁻/⁻^ mouse studies demonstrate that GR plays critical roles in survival and physiological homeostasis [6], [26]. Beyond transactivation, GR also mediates transrepression through nGRE binding and indirect transcriptional regulation via interactions with other transcription factors. In addition to these genomic pathways, GCs elicit rapid non-genomic effects through cytosolic and membrane-bound GRs, as well as non-specific membrane interactions [27]. Furthermore, GC-GR signaling exerts epigenetic effects by modulating histone modifications, DNA and RNA methylation, which can even be transmitted across generations; these mechanisms are discussed in detail in transgenerational epigenetic inheritance of GC-induced effects.

GCs regulate multiple physiological processes, metabolic homeostasis, anti-inflammatory responses, immune function, and electrolyte balance, via genomic (direct and indirect), epigenetic, and non-genomic mechanisms. This review focuses on GC-mediated metabolic regulation in glucose and lipid metabolism, examines diseases linked to dysregulated GC activity, and discusses current therapeutic strategies, remaining challenges, and future directions.

## 4 Crosstalk between glucocorticoids and other endocrine systems

GCs interact with other endocrine systems, including the hypothalamic-pituitary-thyroid (HPT) axis and the hypothalamic-pituitary-gonadal (HPG) axis, through extensive crosstalk with the HPA axis. Elevated GC concentrations inhibit thyroid axis function through a multi-faceted mechanism involving suppression of thyroid-stimulating hormone (TSH) release, attenuation of pituitary TSH responsiveness to thyrotropin-releasing hormone stimulation, and augmentation of T3-mediated negative feedback inhibition of TSH secretion [28]. These coordinated effects typically result in reduced circulating T3 levels, while thyroxine concentrations remain largely unchanged or show only modest reductions [28]. This discrepancy primarily reflects GC-mediated inhibition of peripheral deiodinase activity, which suppresses the conversion of T4 to the more biologically active T3 [29]. Thyroid dysfunction reciprocally affects HPA axis function through bidirectional regulation. Elevated thyroid hormone concentrations enhance the adrenal sensitivity to ACTH and accelerate HPA axis maturation, thereby augmenting GC production [30]. Conversely, thyroid hormone deficiency suppresses both ACTH secretion and adrenal GC synthesis [31]. These reciprocal interactions underscore the integrated nature of endocrine regulation and demonstrate how dysregulation in one axis inevitably compromises the function of interconnected systems [31].

The HPA and HPG axes exhibit reciprocal functional interactions that coordinate stress responses with reproductive physiology. Elevated GC levels suppress the HPG axis by attenuating gonadotropin-releasing hormone (GnRH) pulse secretion from the hypothalamus, inhibiting the release of gonadotropins (luteinizing hormone and follicle-stimulating hormone) from the anterior pituitary, and consequently downregulating gonadal steroidogenesis, through both direct GR signaling in hypothalamic and pituitary cells, as well as indirect effects on neurotransmitter systems that regulate GnRH neurons [32]. However, the extent to which GCs impair reproductive performance is highly context-dependent, contingent upon species differences, the chronicity of exposure, and baseline endocrine status [33]. Additionally, sex hormone-mediated modulation of the HPA axis, arising from distinct patterns of sex hormone receptor expression in neuroendocrine circuits, alters GC secretion and sensitivity, further manifesting as sexual dimorphism in clinical metabolic disorders. The paraventricular nucleus (PVN) expresses abundant estrogen receptors but limited androgen receptors, such that estradiol directly modulates PVN neurons while androgens regulate the HPA axis indirectly via the medial preoptic area and septal regions [34], [35]. Estradiol substantially enhances GC secretion through coordinated mechanisms across hypothalamus, pituitary, and adrenal levels, as evidenced by marked decline in stress-induced ACTH and corticosterone following ovariectomy and their restoration with estradiol replacement [36]. A critical mechanism underlying these effects involves estradiol's impairment of GR-mediated negative feedback at the pituitary level, reducing corticotroph sensitivity to circulating GC and thereby disinhibiting GC secretion [37]. Additionally, estradiol upregulates circulating CBG levels, amplifying total plasma cortisol concentration [35]. In striking contrast, androgens exert antagonistic effects on HPA axis function, suppressing GC secretion and signaling at each regulatory level. The detailed mechanisms of sex hormone-mediated regulation of GC are summarized in Table 1 [35], [36], [37], [38], [39], [40], [41].

**Table 1:** Key features of sex hormone effects on the HPA axis.

**Feature**	**Estradiol**	**Androgens**	**references**
Overall action	Enhancement	Suppression	[36]
PVN	CRH/AVP↑	CRH/AVP↓	[35]
Anterior pituitary	ACTH↑; GR Feedback↓	ACTH↓; GR feedback↑	[40]
Adrenal cortex	Enhanced ACTH sensitivity	Degraded ACTH sensitivity	[37]
CBG levels	Upregulation	Downregulation	[38], [41]
HPA axis activity	Elevated (highly responsive)	Reduced (constrained)	[35]
Stress susceptibility	High	Low	[39]
Clinical consequences	Increased susceptibility to HPA axis hyperactivation disorders	Reduced HPA hyperactivation risk, but severe systemic complications occur when dysregulation occurs	[36]

HPA. Hypothalamic-pituitary-adrenal; PVN. Paraventricular nucleus; CRH. Corticotropin-releasing hormone; AVP. Arginine vasopressin; ACTH. Adrenocorticotropic hormone; CBG. Corticosteroid-binding globulin

## 5 Metabolic functions of glucocorticoids

GCs play critical roles in maintaining energy homeostasis, functioning as key metabolic messengers that regulate transcription of numerous metabolism-related genes. These effects are particularly evident in organs directly involved in energy metabolism, including the pancreatic islets, skeletal muscle, liver, and adipose tissue.

### 5.1 Pancreas islets

**Fig. 3:**
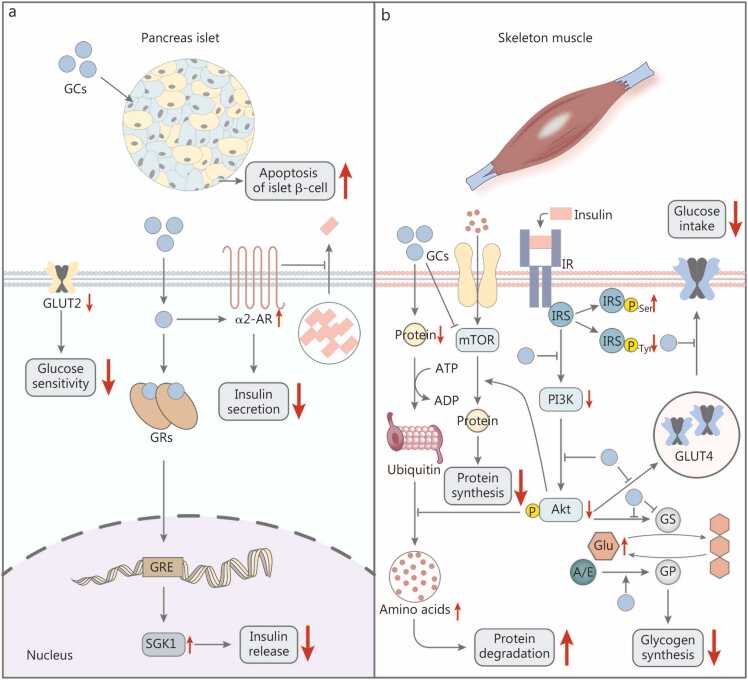
The metabolic effects of glucocorticoids. The metabolic functions of GCs in the pancreas (**a**) and skeletal muscle (**b**). GC. Glucocorticoid; GLUT. Glucose transporter; α2-AR. α2-adrenergic receptor; GR. Glucocorticoid receptor; GRE. Glucocorticoid-response element; SGK1. Serum- and glucocorticoid-inducible kinase 1; mTOR. Mammalian target of rapamycin; IR. Insulin receptor; IRS. Insulin response substrate; PI3K. Phosphoinositide 3-kinase; Akt. Protein kinase B; Glu. Glucose; A/E. Adrenaline/epinephrine; GS. Glycogen synthesis; GP. Glycogen phosphorylase.

As a key regulator of energy homeostasis, GCs exert dose-dependent and context-dependent effects on pancreatic islet function, modulating insulin secretion to meet metabolic demands. Physiologically, GCs support insulin secretion by enhancing cyclic adenosine monophosphate (cAMP) signaling [42]. However, excessive GC exposure inhibits this pathway and suppresses insulin secretion from the islets [43]. And GC-GR signaling impairs insulin secretion through multiple coordinated mechanisms. First, GC-GR signaling promotes β-cells apoptosis by downregulating pancreatic-duodenal homeobox 1 (PDX1), a transcription factor essential for pancreatic development and β-cell function [44]. Second, GC-GR signaling upregulates serum- and glucocorticoid-inducible kinase 1 (SGK1) expression, leading to enhanced K^+^ channel activity and reduced Ca^2+^ channel activity and Ca^2+^ influx, ultimately impairing insulin secretion [45]. Concurrently, GC signaling increases α2-adrenergic receptors (α2-ARs) density in islet cells [46], and together with the elevated SGK1 expression, may converge to inhibit insulin release from β-cells [45], [46]. Furthermore, GCs reduce glucose transporter 2 (GLUT2) expression and protein half-life, thereby decreasing GLUT2 levels and impairing both β-cell glucose sensing and glucose-stimulated insulin secretion [47]. In summary, GCs inhibit pancreatic insulin secretion through multiple mechanisms: β-cell apoptosis, K^+^ and Ca^2+^ channel dysregulation, and impaired glucose sensing (Fig. 3**a**).

### 5.2 Skeletal muscle

Skeletal muscle accounts for approximately 80% of whole-body glucose uptake and oxidation under insulin-stimulated conditions, playing a critical role in systemic glucose homeostasis [48]. GCs directly impair skeletal muscle metabolism through three coordinated mechanisms. First, GCs inhibit GLUT4 translocation to the cell membrane, thereby reducing glucose uptake and glycogen storage in skeletal muscle [49]. This is further compounded by GC-mediated disruption of the insulin signaling cascade, including increased serine phosphorylation and decreased tyrosine phosphorylation of insulin receptor substrates (IRS), inhibition of phosphatidylinositol 3-kinase (PI3K) activity, and reduction of pAKT levels, collectively impairing insulin sensitivity and glucose utilization [50]. Second, GCs dysregulate glycogen metabolism by inhibiting glycogen synthesis while promoting glycogen phosphorylase activation, thereby enhancing glycogenolysis and raising blood glucose levels [51]. These coordinated effects collectively impair glucose utilization in skeletal muscle, diminishing oxidative glucose metabolism and ultimately contributing to muscle insulin resistance. Third, GCs suppress protein synthesis while enhancing ubiquitin-dependent proteolysis through upregulation of key muscle-specific E3 ubiquitin ligases, including MuRF1 and MAFbx, via both direct transcriptional activation and indirect antagonism of insulin-mediated anabolic signaling [52]. This GC-driven shift toward net protein catabolism in skeletal muscle not only leads to muscle wasting but also elevates circulating amino acid levels, which are subsequently transported to the liver to serve as gluconeogenesis substrates, further contributing to hyperglycemia [53]. Therefore, GCs orchestrate skeletal muscle metabolism by modulating glucose uptake, glycogen turnover, and protein catabolism (Fig. 3**b**).

### 5.3 Adipose tissue

The effects of GCs on adipose tissue are profoundly tissue-specific and exhibit marked sexual dimorphism. The human body contains three major adipose compartments: subcutaneous adipose tissue (SAT), visceral adipose tissue (VAT), and brown adipose tissue (BAT), which respond distinctly to GC exposure [54].

In SAT, GCs induce tissue-level insulin resistance and promote lipolysis, yet the metabolic consequences differ markedly between sexes. Women preferentially accumulate SAT, particularly in subcutaneous depots of the hip and thigh regions, where estrogenic effects enhance SAT expandability and metabolic benignity [55]. This SAT-predominant phenotype in women provides substantial metabolic buffering against severe systemic insulin resistance, even in the presence of GC-induced local insulin resistance [55]. In contrast, men preferentially accumulate VAT rather than SAT, resulting in greater metabolic vulnerability, as discussed in the following section.

In striking contrast to SAT, GCs promote VAT expansion, adipocyte hypertrophy, and ectopic lipid deposition, driving metabolic remodeling [56]. This metabolic remodeling is further compounded by VAT's heightened sensitivity to GC-stimulated lipolysis, which triggers chronic inflammation, abundant proinflammatory cytokine production, and reduced adiponectin secretion [57]. The increased free fatty acid flux from VAT lipolysis directly fuels hepatic gluconeogenesis and lipogenesis, creating a vicious cycle of hyperglycemia, dyslipidemia, and ectopic lipid deposition [58]. Men preferentially accumulate VAT, a distribution that translates into greater metabolic vulnerability with more severe insulin resistance and metabolic syndrome at lower cumulative GC doses than women [59]. Post-menopausal women, losing estrogen’s protective influence on SAT maintenance and VAT limitation, progressively acquire a male-like metabolic phenotype with increased VAT burden and substantially elevated metabolic disease risk [60].

BAT plays a critical counter-regulatory role in energy expenditure and thermogenesis [61]. GC excess impairs BAT thermogenic function through multiple coordinated mechanisms [62]. Women generally maintain higher BAT activation capacity, contributing to their metabolic advantage under GC excess, whereas men exhibit lower BAT-mediated thermogenesis, exacerbating their metabolic vulnerability [63], [64].

Collectively, GC-induced effects across these three adipose tissue compartments, SAT insulin resistance, VAT expansion with inflammatory activation, and BAT thermogenic impairment, lead to profound adipose tissue redistribution and maladaptive metabolic reprogramming; the detailed tissue-specific mechanisms and associated sexual dimorphism are summarized in Table 2 [55], [57], [59], [65], [66], [67], [68], [69], [70], [71], [72], [73].

**Table 2:** Tissue-specific effects of glucocorticoids on adipose tissue and sexual dimorphism.

**Parameter**	**SAT**	**VAT**	**BAT**	**References**
Insulin signaling	IRS↓; Vit D receptor↑; PI3K/Akt phosphorylation↓	SREBP-1c↑; FAS↑	Pgc1α↓	[65], [66], [67]
Glucose uptake	GLUT4 translocation↓; Glucose disposal↓	Glucose uptake impairment; Insulin resistance↑	Maintained; Independent of insulin	[68]
Lipogenesis	Insulin-stimulated lipogenesis↓	Triglyceride accumulation↑	-	[69], [70]
Lipolysis	HSL, ATGL↑; FFA release↑	Highly sensitive; Massive FFA→liver	Minimal lipolytic response	[70]
Thermogenesis	No thermogenic activity	Browning↓; Thermogenic potential↓	Brown adipocyte whitening; UCP1↓; Thermogenesis↓	[71], [72]
Adipogenesis	Minimal expansion	LMO3↑; Adipocyte hypertrophy; Depot expansion↑	Minimal expansion	[73]
Inflammation	Relatively anti-inflammatory	Chronic inflammation; IL-6, TNF-α, MCP-1↑; Adiponectin↓	Anti-inflammatory; Minimal cytokine production	[57]
Sexual dimorphism				
Female	SAT predominance; Estrogenic protection; Metabolic buffering	Lower VAT; Estrogenic suppression	Higher BAT activation	[55]
Male	Lower SAT; Reduced buffering	VAT predominance; Early metabolic dysfunction	Lower BAT activation; Thermogenesis↓	[59]

↑. Upregulation/Increase; ↓. Downregulation/Decrease; “-”. Not applicable; SAT. Subcutaneous adipose tissue; VAT. Visceral adipose tissue; BAT. Brown adipose tissue; IRS. Insulin receptor substrate; PI3K. Phosphatidylinositol 3-kinase; Akt. Protein kinase B; GLUT4. Glucose transporter type 4; HSL. Hormone-sensitive lipase; ATGL. Adipose triglyceride lipase; FFA. Free fatty acid; SREBP-1c. Sterol regulatory element-binding protein 1c; FAS. Fatty acid synthase; UCP1. Uncoupling protein 1; Pgc1α. Peroxisome proliferator-activated receptor gamma coactivator 1-alpha; LMO3. LIM domain only 3; SNS. Sympathetic nervous system

### 5.4 Liver

The liver serves as a critical metabolic hub orchestrating whole-body energy homeostasis. GC excess profoundly alters hepatic glucose and lipid metabolism through tissue-specific mechanisms that differ between physiologic and pathologic conditions [74].

During fasting, starvation, or intense physical activity, GC-mediated gluconeogenesis and hepatic lipid metabolism represent adaptive responses [75]. GCs potently stimulate hepatic gluconeogenesis by antagonizing insulin action and synergizing with counter-regulatory hormones (adrenaline and glucagon), thereby maintaining blood glucose levels for cerebral function and erythropoiesis [76]. Simultaneously, GC signaling in hepatic resident macrophages suppresses tumor necrosis factor-α (TNF-α) and promotes acute fasting-adapted lipid handling through enhanced hepatic ketogenesis while restraining lipogenic transcription [77]. The molecular mechanisms underlying GC-induced hepatic gluconeogenesis, lipogenesis, and their condition-dependent metabolic consequences are summarized in Table 3 [76], [78], [79], [80], [81], [82]. However, the critical physiological transition occurs under pathologic conditions of chronic GC excess, where these adaptive mechanisms become maladaptive [78]. Chronic GC exposure drives persistent hepatic insulin resistance, sustained hepatic fatty acid uptake, and impaired mitochondrial β-oxidation, collectively promoting hepatic triglyceride accumulation and the development of metabolic dysfunction-associated steatotic liver disease (MASLD) [78]. Consequently, the combination of elevated hepatic glucose production and hepatic lipid accumulation creates a pathogenic state of simultaneous hyperglycemia and dyslipidemia, contributing to metabolic syndrome and cardiovascular disease [79].

**Table 3:** Glucocorticoid (GC)-induced hepatic metabolic regulation: gluconeogenesis, lipogenesis and steatosis, and condition-dependent metabolic outcomes.

**Parameter**	**Hepatic gluconeogenesis**	**Hepatic lipogenesis and steatosis**	**References**
Insulin action	GCs antagonize insulin; Suppress insulin-mediated glucose suppression; Promote hepatic insulin resistance	Insulin resistance; FFA uptake sustained; β-oxidation impaired	[76]
Hormonal interaction	GCs synergize with adrenaline and glucagon	Inhibits TNF-α; Promotes ketogenesis	[80]
Key enzymes/regulators	PEPCK↑; G6Pase↑; Klf9↑→Pgc1α↑	SETDB2↑→Insig2a↑; SREBP-1c↓	[81], [82]
Substrate availability	Amino acids↑ (from muscle); FFAs↑ (from adipose tissue)	Hepatic FFA uptake↑; Triglyceride accumulation↑	[76]
Metabolic outcomes by condition			
Physiologic conditions (fasting, exercise)	Adaptive; Maintains blood glucose; Supports brain function and erythropoiesis	Adaptive; Reduced lipogenesis via SETDB2/Insig2a; Promotes ketogenesis	[80], [82]
Chronic GC excess (pathologic)	Maladaptive; Persistent hyperglycemia; Impaired glucose tolerance; Insulin resistance; T2DM risk↑	Maladaptive; Hepatic triglyceride↑; MASLD development; Dyslipidemia↑	[78], [79]

↑. Upregulation/Increase; ↓. Downregulation/Decrease; PEPCK. Phosphoenolpyruvate carboxykinase; G6Pase. Glucose-6-phosphatase; Klf9. Krüppel-like factor 9; Pgc1α. Peroxisome proliferator-activated receptor gamma coactivator 1-alpha; SETDB2. SET domain bifurcated histone lysine methyltransferase 2; Insig2α. Insulin-induced gene 2α; SREBP-1c. Sterol regulatory element-binding protein 1c; GR. Glucocorticoid receptor; TNF-α. Tumor necrosis factor-α; FFA. Free fatty acid; T2DM. Type 2 diabetes mellitus; MASLD. Metabolic dysfunction-associated steatotic liver disease;

Fundamentally, the paradoxical nature of GC-induced hepatic metabolism reflects the hormone’s dual role, promoting survival during acute stress through adaptive metabolic reprogramming, yet driving severe metabolic pathology when dysregulation occurs chronically. The transition from adaptive to maladaptive hepatic metabolism, combined with GC-induced glucose and lipid metabolic dysfunction, contributes to systemic metabolic dysregulation.

## 6 Transgenerational epigenetic inheritance of GC-induced effects

Beyond immediate physiological responses, fetal exposure to excessive GCs exerts lasting epigenetic programming effects on metabolism, stress responsiveness, and behavior [83]. Notably, these effects are not confined to the directly exposed individual but can be transmitted through both maternal and paternal lineages to subsequent generations [84]. Under physiological conditions, the placenta maintains a substantial gradient between maternal and fetal GC levels primarily via 11β-HSD2, which inactivates cortisol to cortisone [83]. However, this fetal protection is inherently incomplete even under basal conditions and can be further compromised by maternal undernutrition or chronic stress, both of which suppress 11β-HSD2 expression and activity [85], [86]. Consequently, these physiological and environmental factors collectively dictate the degree of fetal GC exposure, especially under conditions of elevated maternal levels that overwhelm the placental enzymatic barrier [85]. Prenatal GC overexposure is associated with adverse outcomes, including low birth weight, prematurity, and intrauterine growth restriction, all established risk factors for adult metabolic disease [87]. In the F1 generation, offspring demonstrate multiple metabolic disturbances such as obesity, hyperinsulinemia, insulin resistance, hypertension, metabolic syndrome, upregulated hepatic GR and PEPCK expression, and heightened HPA axis activity [88], [89], [90]. Intriguingly, similar transgenerational impacts, including impaired memory and metabolic dysregulation, are observed in offspring following paternal stress or synthetic GC exposure prior to mating [91].

The molecular continuity of these persistent effects is primarily sustained by germline epigenetic reprogramming. GCs modulate DNA methylation patterns by binding to GREs in gene promoters [22]. For instance, increased methylation of the *NR3C1* promoter in the hypothalamus attenuates negative feedback regulation [92], while hypermethylation of the *Pgc1α* promoter in fetal BAT contributes to metabolic dysfunction [93]. Furthermore, GC-induced alterations in sperm microRNA expression, such as the downregulation of miR-466b-3p, further drive metabolic and developmental abnormalities in offspring [94], [95].

The most distinctive aspect of this phenomenon is the extension of these effects into transgenerational inheritance (F3 and beyond). Unlike intergenerational effects (F1-F2), which involve direct exposure of the fetus or its developing germ cells, modifications persisting into the F3 generation represent a stable transmission of epigenetic memory [96]. Adult F3 offspring prenatally exposed to GCs display altered gene expression in the PVN of the hypothalamus and persistent reproductive dysfunction [96], [97]. It should be noted, however, that transgenerational epigenetic data are derived predominantly from rodent models, and direct human evidence for such multigenerational inheritance remains limited. These findings highlight an important outstanding question: the timing and mechanisms by which epigenetic modifications are erased across generations.

## 7 Clinical manifestations of glucocorticoid homeostatic imbalance

**Fig. 4:**
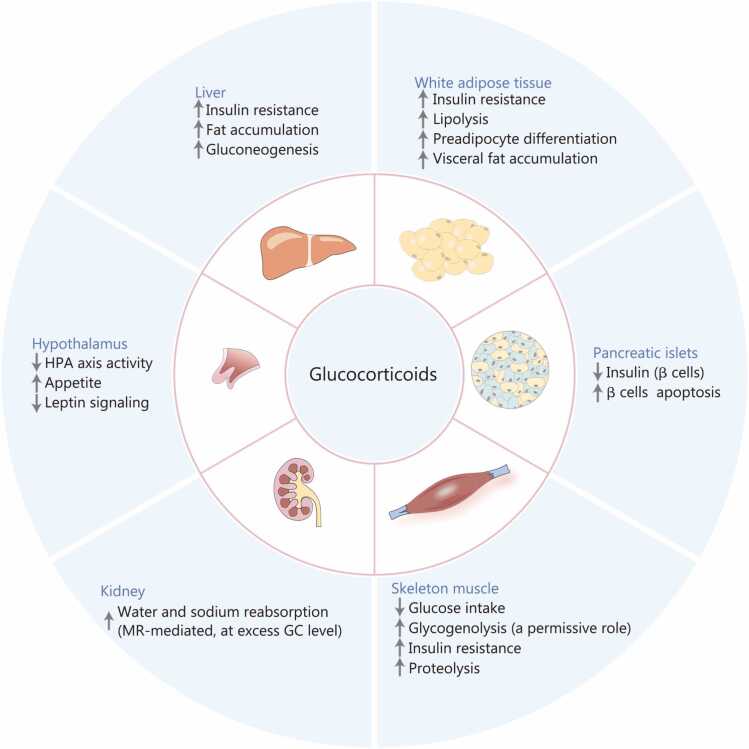
Target tissues and metabolic activities of glucocorticoids. Glucocorticoids (GCs) promote glycogenolysis in skeletal muscle indirectly through a permissive mechanism, by upregulating glucagon and epinephrine receptor sensitivity rather than directly stimulating glycogen breakdown. HPA. Hypothalamic-pituitary-adrenal; MR. mineralocorticoid receptors.

GC excess has profound systemic consequences in affected patients, manifesting as a spectrum of clinical metabolic disorders [98]. Table 4 [99], [100], [101], [102], [103], [104], [105], [106], [107], [108], [109], [110] comprehensively summarizes the multi-organ health effects and complications of GC excess, highlighting the underlying pathogenic mechanisms and notable sexual dimorphism in disease manifestations and severity. Disruptions in GC homeostasis, spanning from pathological excess to glandular insufficiency, precipitate a broad spectrum of metabolic and systemic dysfunctions. This is clinically manifested through contrasting conditions such as Cushing’s syndrome and Addison’s disease, which represent opposite ends of the GC functional spectrum. Given the systemic nature of these disorders, we further explore how GCs specifically modulate carbohydrate metabolic pathways to induce these observed physiological changes (Fig. 4).

**Table 4:** Systemic health effects and complications of glucocorticoid excess: pathogenic mechanisms and sexual dimorphism.

**Conditions**	**Pathogenic mechanism**	**Clinical features**	**Female characteristics**	**Male characteristics**	**References**
Cushing’s syndrome	ACTH overproduction; Elevated GC	Central obesity; Proximal weakness; Purple striae; Hyperglycemia; Hypertension; Mood disorders	Higher incidence; Earlier diagnosis	Lower incidence; Greater diagnostic challenges	[100], [106], [107]
Metabolic syndrome	Central obesity; Insulin resistance; Dyslipidemia	Abdominal obesity; Elevated glucose; Elevated Triglycerides; Reduced HDL; Hypertension	Later onset; Pre-menopausal protection from estrogen; Rapid increase post-menopause as VAT burden increases	Earlier and more severe central obesity; Sustained dyslipidemia; Persistent dominant atherogenic lipid profile	[99], [108]
Diabetes	Insulin resistance; Impaired glucose tolerance; Elevated hepatic gluconeogenesis	Fasting hyperglycemia; Impaired glucose tolerance; Insulin resistance	Lower diabetes risk; Better maintained hepatic insulin sensitivity; Reduced risk due to estrogen	Higher diabetes risk; More severe hyperglycemia; Earlier diabetes onset	[101], [102]
MASLD	Increased hepatic lipid accumulation; Impaired β-oxidation; Enhanced lipogenesis	Hepatic steatosis; Elevated transaminases; Risk of fibrosis	Lower overall MASLD prevalence; Slower steatosis accumulation, but higher advanced fibrosis risk once disease is established, especially post-menopause	Higher overall MASLD incidence and prevalence; Faster early disease progression; Testosterone-mediated effects are complex	[103], [104], [109]
Cardiovascular disease	Systemic inflammation; Endothelial dysfunction; Prothrombotic state	Coronary artery disease; Myocardial infarction; Heart failure; Ischemic stroke; Arrhythmias	Pre-menopausal protection; Improved lipid profile; Rapid CVD increase post-menopause as GC excess persists	Earlier CVD onset, higher event rates, and greater severity at equivalent GC doses; Sustained high risk with age	[105]
Hypertension	Sodium retention; Endothelial dysfunction; Vascular inflammation	Elevated blood pressure; Impaired diastolic function	Lower prevalence; Pre-menopausal protection; Rapid onset with GC excess; Loss of estrogen’s vasodilatory effect post-menopause	Higher Prevalence; Greater severity; Sustained hypertension with advancing GC excess	[110]

GC. Glucocorticoid; ACTH. Adrenocorticotropic hormone; FAO. Fatty acid oxidation; CVD. Cardiovascular disease; VAT. Visceral adipose tissue; HDL. High-density lipoprotein; MASLD. Metabolic dysfunction-associated steatotic liver disease

### 7.1 Cushing’s syndrome and obesity

Cushing’s syndrome arises from prolonged exposure to excess GCs, either through exogenous administration or endogenous overproduction. Endogenous CS results from three primary causes: pituitary ACTH-secreting adenomas, ectopic ACTH secretion from non-pituitary tumors, and cortisol-secreting adrenal tumors [111]. Clinically, patients with Cushing’s syndrome typically present with distinctive physical features, including buffalo hump, moon face, facial acne, hirsutism, and broad purple striae, as well as preferential fat redistribution to visceral depots, which increases systemic inflammation [100], [106]. Crucially, Cushing’s syndrome induces multiple metabolic abnormalities, with visceral obesity, hypertension, and impaired glucose metabolism affecting a substantial proportion of patients [112]. Compared to patients with nonfunctioning adrenal tumors, those with Cushing’s syndrome exhibit significantly higher prevalence and severity of type 2 diabetes mellitus (T2DM) [113]. Cardiovascular complications arise primarily through accelerated atherosclerosis driven by dyslipidemia, hypertension, and hyperglycemia, while infectious complications result from GC-mediated immunosuppression and impaired immune surveillance. Together, these cardiovascular and infectious complications represent the leading causes of death in Cushing's syndrome patients [114].

The mechanistic link between GCs and adiposity is driven by both central and peripheral pathways. Centrally, elevated GCs enhance neuropeptide Y expression in the arcuate nucleus, promoting hyperphagia and increased caloric intake [115]. Peripherally, GCs facilitate visceral fat accumulation by upregulating Klf9 in macrophages and modulating glucose-lipid metabolism across the liver and adipose tissue [116]. Clinically, hair cortisol concentrations demonstrate a strong positive correlation with waist circumference, with elevated hair cortisol predicting central adiposity and obesity risk up to a decade later [117], [118]. Consistent with a causal role of GCs in obesity, animal studies demonstrate that bilateral adrenalectomy reduces food intake and weight gain, preventing obesity development, an effect abolished by cortisol replacement [119]. Notably, circulating cortisol levels in common obesity do not always mirror increased adiposity, as plasma levels may remain normal or even low. This discrepancy is often explained by increased cortisol clearance or altered local 11β-HSD1 activity [120]. Specifically, elevated 11β-HSD1 expression in obese adipose tissue amplifies local GC signaling despite normal systemic concentrations. This local hypercortisolism further exacerbates GC-driven lipid accumulation and insulin resistance in adipose tissue, thereby sustaining and exacerbating the obese phenotype [19].

### 7.2 Metabolic syndrome

Metabolic syndrome **(**MetS) represents a cluster of interrelated metabolic abnormalities, including central obesity, hypertension, hyperglycemia, insulin resistance, and dyslipidemia, that collectively elevate cardiovascular disease risk. GC excess positively correlates with MetS pathogenesis by simultaneously driving dysfunction across multiple metabolic axes. At the adipose tissue level, GC-induced preferential visceral fat accumulation increases free fatty acid flux, fueling hepatic insulin resistance and dyslipidemia [108]. In the liver, GC-driven upregulation of gluconeogenic enzymes and impaired insulin signaling contribute to persistent hyperglycemia and hepatic steatosis [99]. In skeletal muscle, GC-mediated insulin resistance reduces peripheral glucose disposal, further exacerbating systemic hyperglycemia. In the pancreas, GC-induced β-cell dysfunction impairs compensatory insulin secretion, accelerating the progression from insulin resistance to overt type 2 diabetes [99]. Notably, elevated circulating GC levels are not universally observed in MetS, potentially reflecting enhanced GC clearance mediated by 5α-reductase and 5β-reductase, which accelerate the hepatic inactivation and urinary excretion of GC [106]. Beyond systemic GC clearance, local 11β-HSD1 expression plays a pivotal role by regenerating active cortisol from inactive cortisone in metabolically active tissues. This tissue-specific GC amplification accelerates MetS onset and progression; accordingly, *11β-HSD1* knockout mice display significant protection from MetS development [108].

### 7.3 Diabetes

Diabetes results from insulin resistance coupled with impaired insulin secretion, together causing hyperglycemia and impaired glucose utilization [121]. Chronic hyperinsulinemia has been proposed as a primary T2DM trigger, whereby sustained insulin hypersecretion induces insulin resistance, ultimately culminating in β-cell dysfunction and decompensated secretion [122]. Elevated GCs increase blood glucose, initially triggering compensatory insulin secretion, but ultimately leading to T2DM [122]. Population-based studies revealed robust associations between higher serum cortisol (within the physiologic range) and reduced insulin secretion capacity [123].

The clinical impact of GC-induced hyperglycemia is well documented. Exogenous GC therapy in non-diabetic patients substantially increases hyperglycemia and diabetes risk, with prolonged exposure (>1 month) particularly problematic, as evidenced by hyperglycemia incidence exceeding 50% among hospitalized non-diabetic patients receiving glucocorticoid therapy (blood glucose ≥200 mg/dl) [124] and hyperglycemia emerging as the most frequent adverse event in a multicenter randomized controlled trial of acute respiratory distress syndrome patients treated with dexamethasone [125]. These patterns persist across different clinical scenarios, including sepsis patients who demonstrate elevated hyperglycemia risk with GC treatment compared to controls [126].

Although GCs extensively regulate glucose metabolism, significant gaps in the complete molecular mechanisms remain to be fully elucidated, despite the identification of several key genes and molecular interactions involved in these processes. In obese individuals, elevated hepatic Cyp17a1 expression increases 17-hydroxyprogesterone levels, activating GR signaling and promoting hyperglycemia and insulin resistance [127]. In experimental rat models, prenatal DEX exposure impaired pancreatic β-cell function via GR-mediated histone H3 deacetylation at the angiotensin-converting enzyme 2 (*ACE2*) gene promoter, thereby downregulating ACE2 expression [128]. Moreover, GC-GR signaling upregulates Gadd45β and hepatic Klf9, which activate Pgc1α-mediated hepatic gluconeogenesis and elevate blood glucose [81], [129].

In T2DM, elevated blood glucose thresholds trigger counter-regulatory hormone secretion, including GC, with counter-regulatory hormone release thresholds remaining elevated, a physiological adaptation that potentially protects against severe hypoglycemia [130]. At the central level, within the central nervous system, insulin synergizes with neuropeptide Y to suppress appetite, whereas GCs promote food intake by inhibiting blood-brain barrier insulin transport, which disrupts central appetite regulation [131]. At the peripheral level, GCs upregulate SGK1 in adipose tissue, reducing Akt phosphorylation and IRS expression, ultimately causing insulin resistance [132]. Furthermore, insulin-resistant individuals demonstrate increased skeletal muscle *GR* mRNA expression, enhancing GC sensitivity and exacerbating insulin resistance [133]. At the genetic level, the GR rs9324924 polymorphism modulates T2DM risk, particularly in individuals with elevated serum cortisol [134]. Together, these mechanisms establish a bidirectional vicious cycle. HPA axis alterations and elevated GCs promote insulin resistance, while reciprocally, impaired insulin secretion and persistent hyperglycemia modulate HPA axis activity and GC secretion, perpetuating disease progression. Clinically, endogenous hypercortisolism is present in 23.8% (95% CI 21.3–26.5; *n*=1057) of patients with refractory diabetes who remain poorly controlled despite multiple antidiabetic medications, as identified by the 1-mg overnight dexamethasone suppression test in the multicentre cross-sectional CATALYST study [135]. Furthermore, administering the GR antagonist mifepristone to these patients leads to a substantial decline in glycated hemoglobin [136]. This underscores cortisol-targeted therapy as a potent personalized approach for managing refractory diabetes associated with hypercortisolism.

### 7.4 Metabolic dysfunction-associated steatotic liver disease

MASLD is the most common liver disease worldwide, which affects more than one-third of adults [137]. MASLD pathogenesis is influenced by various factors encompassing genetic, metabolic, and endocrine mechanisms [104]. Genome-wide association studies have identified several genetic variants associated with MASLD susceptibility and disease severity. Key among these are single-nucleotide polymorphisms in *PNPLA3* and *TM6SF2*, which increase disease risk. Conversely, loss-of-function variants in *HSD17B13*, particularly *rs72613567*, have emerged as protective factors that reduce MASLD risk and slow progression from hepatic steatosis to steatohepatitis [138]. Beyond genetic predisposition, metabolic dysregulation, including insulin resistance, dyslipidemia, and obesity-driven lipid accumulation, also plays a central role in MASLD development and progression [104].

Endocrine dysfunction plays a critical role in MASLD pathogenesis. The link between hypercortisolism and MASLD is largely mediated by the accumulation of visceral adiposity, a well-established independent risk factor for hepatic steatosis. In patients with Cushing’s syndrome, for instance, the prevalence of hepatic steatosis reaches approximately 20%, as assessed by CT using a liver-to-spleen attenuation ratio, with the severity of steatosis directly correlating with the extent of visceral fat [109]. It should be noted, however, that CT may underestimate the true prevalence of hepatic steatosis compared with MRI or liver biopsy. This underscores the role of chronic GC elevation in driving ectopic lipid deposition within the liver. DEX is commonly used as replacement therapy for hypopituitary patients with growth hormone deficiency. In animal models, DEX-treated rats display liver enlargement and substantial hepatic lipid accumulation confirmed histopathologically [139], and liver-specific GR disruption ameliorates hepatic steatosis by normalizing triglycerides through *Hes1* gene upregulation [79], collectively supporting the direct role of hepatic GR signaling in lipid accumulation. Mouse models with hepatic GR deficiency exhibit improved hepatic steatosis [79]. Mechanistically, GCs promote hepatic de novo lipogenesis and enhance triglyceride synthesis while simultaneously suppressing fatty acid β-oxidation [140]. Additionally, GCs increase hepatic lipid availability by promoting adipose tissue lipolysis. DEX upregulates lipoprotein lipase mRNA expression in adipocytes, increasing plasma free fatty acid levels that are subsequently transported to the liver for lipogenesis [141]. This lipolytic effect is further amplified through Angptl4 signaling, whereby DEX-induced Angptl4 expression in white adipose tissue stimulates additional lipolysis via cAMP pathway activation, as evidenced by marked reductions in DEX-induced hypertriglyceridemia and hepatic steatosis in *Angptl4* knockout mice [142]. At the transcriptional level, GC-GR signaling activates the *Fto* gene, which mediates m^6^A mRNA demethylation of adipogenesis-related transcripts and promotes lipid accumulation; *Fto* knockout notably attenuates GC-induced hepatic steatosis, confirming its mechanistic role [143]. Furthermore, elevated GCs increase glucose and insulin concentrations, stimulating sterol regulatory element-binding protein 1c and carbohydrate response element-binding protein expression and driving lipogenic gene expression and hepatic lipid deposition [140].

Beyond direct metabolic effects, GCs alter hepatic immune and transcriptional regulation to promote steatosis and inflammation through distinct mechanisms. At the immune level, obese individuals demonstrate attenuated GR-GILZ axis signaling in hepatic Kupffer cells, the resident macrophages of the liver responsible for maintaining hepatic immune homeostasis [144]. This attenuation compromises their anti-inflammatory capacity, shifting Kupffer cells toward a pro-inflammatory phenotype that promotes hepatic inflammation and contributes to fatty liver disease progression [144].

### 7.5 Cardiovascular disease

GCs exert profound effects on cardiovascular function and disease risk through multiple mechanisms, including systemic inflammation, endothelial dysfunction, and promotion of a prothrombotic state, contributing to clinical manifestations such as hypertension and dyslipidemia [105]. Cushing’s syndrome exemplifies the severe cardiovascular consequences of chronic GC excess, with patients exhibiting substantially elevated mortality compared to unaffected individuals, with cardiovascular disease as the predominant cause of death [145]. This dramatically increased cardiovascular risk is largely attributable to GC-driven metabolic dysregulation, as chronic GC excess directly induces visceral obesity, hyperglycemia, and dyslipidemia, all well-established independent cardiovascular risk factors [146].

First, GCs promote very low-density lipoprotein (VLDL) secretion, contributing to atherogenic lipid profiles, increasing atherosclerosis risk [147]. This pathogenic mechanism is supported by functional evidence demonstrating that selective inhibition of 11β-HSD1 significantly reduces hepatic VLDL secretion and attenuates atherosclerotic plaque development in ApoE-null mice [148]. Second, GC-GR signaling promotes cardiomyocyte hypertrophy through a mechanism involving suppression of the SRSF4-GAS5 axis. In vitro evidence demonstrates this effect: DEX-treated H9C2 cardiomyocytes exhibit cellular enlargement and upregulation of cardiac hypertrophy markers [149]. The underlying mechanism involves GR signaling-mediated downregulation of the SRSF4-GAS5 axis, which drives ventricular hypertrophy. This molecular mechanism is clinically validated by RNA-seq analysis in Cushing’s syndrome patients, showing decreased *SRSF4* and *GAS5* expression consistent with GR-mediated axis suppression [149]. Furthermore, cardiomyocyte-specific GR-overexpression mice exhibit atrioventricular conduction block, highlighting the adverse cardiac effects of excessive GC signaling on cardiac electrophysiology [150]. The underlying mechanism involves GC-GR signaling-mediated alterations in cardiac ion channel activity, as demonstrated in vitro by its effects on key cardiac currents including I_Na_, I_to,_ and I_Kslow_ [150]. Notably, GCs exhibit cardioprotective properties under specific conditions, including reduction of ischemia-reperfusion myocardial injury [151]. Cardiomyocyte-specific *GR* knockdown mice display early-life cardiac dysfunction, indicating GR signaling importance in cardiac development and function [152]. Furthermore, prednisone attenuates macrophage lipid accumulation and reduces foam cell formation, providing atherosclerotic protection [153]. GC cardiovascular contributions gain further validation from population-based cohort studies. In individuals with immune-mediated disease, dose-dependent cardiovascular risk associations with GC use emerged, even at low doses (<5 mg/d) [154]. Clinicians must carefully monitor cardiovascular function, endogenous cortisol levels, and exogenous GC dosage and duration in treated patients.

### 7.6 Hypertension

Plasma GC concentrations demonstrate a positive correlation with hypertension prevalence, with oral GC use showing dose-dependent associations with hypertension incidence among patients with chronic inflammatory disease [155]. The clinical significance of this dose-dependent relationship is exemplified by rheumatoid arthritis patients receiving prolonged GC treatment, who demonstrate a 17% increased risk of hypertension development [156].

GC-induced hypertension mechanisms have been investigated for decades [157]. Primary GC effects occur in the kidneys and vascular smooth muscle. Excessive cortisol can overwhelm the protective capacity of 11β-HSD2, leading to inappropriate MR activation. This results in potent mineralocorticoid-like effects, such as sodium retention and potassium excretion, which significantly contribute to the development of hypertension [158]. Aldosterone synthase-deficient mice provide evidence that cortisol-MR signaling can compensate for the loss of aldosterone, adequately maintaining water and sodium balance. This is confirmed by the increased water and sodium excretion observed following MR antagonist treatment in these models [159]. Additionally, 11β-HSD2-inactivating mutant mice display hypertension and reduced endothelial nitric oxide (NO) activity, underscoring the critical role of the enzyme in MR activity and vascular tone regulation [160]. These findings suggest GC-MR interaction modulation may represent a GC-induced hypertension therapeutic strategy. Beyond renal effects, GCs contribute to hypertension via direct vascular actions. GCs upregulate angiotensin II type 1 receptor expression in vascular smooth muscle, inhibiting endothelial production of vasodilators, prostacyclin, and NO. This suppression enhances phenylephrine-induced vasoconstriction in endothelium-intact vessels, ultimately elevating blood pressure [161]. Moreover, in rabbit GC-induced hypertension models, vascular smooth muscle cells from aortic rings demonstrate 2-fold increased calcium ion influx versus normotensive controls [110]. Additionally, GR signaling critically suppresses WNK4, a salt-handling kinase, via β2AR-mediated mechanisms, suggesting possible GC involvement in salt-sensitive hypertension [162].

### 7.7 Adrenal insufficiency

Adrenal insufficiency can be classified into primary (PAI), secondary, and GC-induced adrenal insufficiency [163]. PAI results from adrenal gland destruction, developmental dysgenesis, or enzyme deficiencies of either inherited or acquired etiology, manifesting as combined deficiencies in GCs, mineralocorticoids, and androgens [164]. Clinical features range from non-specific early symptoms, including fatigue, weakness, and weight loss, to prominent GC deficiency manifestations such as hypotension, hypoglycemia, and increased infection susceptibility as the disease progresses; distinctly, mineralocorticoid deficiency and compensatory ACTH elevation give rise to salt craving and skin and mucosal hyperpigmentation [164].

In contrast, secondary adrenal insufficiency, which is more prevalent than PAI, stems from insufficient ACTH production, most commonly due to hypothalamic or pituitary pathology (e.g., tumors, trauma) [164], leading to isolated GC insufficiency while usually sparing mineralocorticoid secretion [165]. Another clinically important subtype of adrenal insufficiency is GC-induced adrenal insufficiency, which should be considered in patients who have recently discontinued or tapered supraphysiologic GC doses [166]. This condition develops as a consequence of prolonged supraphysiologic synthetic GC use (e.g., prednisone >5 mg daily for over 3 weeks), which suppresses endogenous CRH and ACTH production via negative feedback [157]. Exogenous GCs activate GR in hypothalamic and pituitary cells, leading to progressive atrophy of the adrenal cortex and reduced steroidogenic capacity. The degree of HPA axis suppression is influenced by GC potency, dose, duration of use, and route of administration, with inhaled and topical GCs also capable of inducing systemic suppression at high doses [166]. Unlike the irreversible nature of many PAI cases, GC-induced adrenal insufficiency offers the potential for HPA axis recovery, necessitating rigorous clinical assessment, such as morning serum cortisol measurement, where levels >300 nmol/L indicate sufficient recovery for safe GC discontinuation [157].

### 7.8 Addison’s disease

Addison’s disease (AD), the most recognized form of PAI, is characterized by insufficient GC and mineralocorticoid production, resulting in life-threatening adrenal crises if untreated [167]. While autoimmune adrenalitis has become the predominant etiology following improved tuberculosis control, the common pathological endpoint across etiologies is destruction of the adrenal cortex and consequent GC deficiency [167]. This chronic GC insufficiency contributes to the significantly elevated mortality risk observed in AD patients, approximately double that of the general population, primarily through increased susceptibility to cardiovascular disease, infections, and adrenal crisis [168].

AD patients frequently present with autoantibodies against 21-hydroxylase, a critical adrenal steroidogenic enzyme, with 86% of AD patients testing positive in a large Norwegian cohort [169]. Additional autoantibodies targeting side-chain cleavage enzyme and 17α-hydroxylase have been identified, collectively reflecting a broad autoimmune attack on the adrenal steroidogenic pathway that ultimately results in GC deficiency [169].

The clinical manifestations of GC imbalance in AD are bidirectional. GC overexposure during replacement therapy produces Cushingoid features, including hypertension, hyperglycemia, and osteoporosis [166], while inadequate GC supplementation during acute stress precipitates adrenal crisis, a life-threatening condition characterized by severe hypotension, hypoglycemia, and cardiovascular collapse that substantially contributes to morbidity and mortality [170]. Conversely, rapid GC reduction or discontinuation precipitates GC withdrawal syndrome, manifesting as fatigue, hypotension, and malaise, reflecting HPA axis suppression and adrenal atrophy induced by chronic GC supplementation [157].

### 7.9 Critical illness-related corticosteroid insufficiency

Critical illness-related corticosteroid insufficiency (CIRCI) is defined as an insufficient endogenous GC response relative to the physiological demands imposed by critical illness, most commonly occurring in patients with trauma, extensive surgery, and septic shock. Unlike classical adrenal insufficiency, CIRCI is not solely characterized by insufficient cortisol production but is instead driven by HPA axis dysregulation, altered GC metabolism, and tissue GC resistance [171].

Regardless of the specific etiology, critical illness disrupts GC homeostasis through HPA axis dysfunction and impaired adrenal GC production [172]. CIRCI manifests clinically as an inadequate GC response to physiological stress, characterized by refractory hypotension unresponsive to fluid resuscitation and vasopressors, persistent systemic inflammation, impaired immune regulation, and hemodynamic instability [172]. The clinical presentation is further complicated by tissue GC resistance, whereby even patients with elevated circulating cortisol levels may exhibit insufficient GC-mediated anti-inflammatory effects due to cytokine-mediated impairment of GR function [173]. Collectively, these manifestations reflect an imbalance between hyperactivated inflammatory pathways and an inadequate or dysregulated GC response.

## 8 Therapeutic options

Synthetic GCs represent vital pharmacological agents with dual roles; they are widely prescribed for allergic diseases, asthma, rheumatoid arthritis, as well as for preventing immune rejection after organ transplantation [174]. However, prolonged exogenous GC administration frequently precipitates an array of metabolic and structural complications, which are further exacerbated by GC resistance commonly induced by high-dose or prolonged exposure. This section reviews current strategies for mitigating the metabolic side effects of GC therapy.

### 8.1 Dose reduction and glucocorticoid resistance

While titrating GCs to the minimum effective dose remains a cornerstone strategy for mitigating systemic complications, its clinical implementation is frequently constrained by GC resistance. This inherent or acquired insensitivity necessitates higher therapeutic doses to achieve adequate anti-inflammatory or immunosuppressive effects, paradoxically amplifying the risk of dose-dependent adverse outcomes [175].

These resistance mechanisms operate across multiple molecular levels. At the receptor level, an elevated GRβ/GRα ratio serves as a key biomarker for clinical insensitivity, as GRβ competitively inhibits active GC signaling [176]. Furthermore, proinflammatory cytokines directly undermine dose-tapering efforts; for instance, IL-17A and TNF-α impair GR-mediated transactivation and transrepression through the PI3K and mitogen-activated protein kinase (MAPK) pathways [177]. Notably, the use of anti-TNF-α agents has been shown to restore GC responsiveness, suggesting that targeting specific inflammatory mediators may facilitate safer dose reduction [177]. At the post-translational level, epigenetic remodeling through histone deacetylase 2 (HDAC2) can reverse GC resistance, while excessive MAPK activation reduces the anti-inflammatory efficacy of existing GR-GC complexes [178], [179]. Targeted inhibition of components like c-Jun N-terminal kinase (JNK) or extracellular signal-regulated kinase (ERK), both of which phosphorylate GR and impair its transcriptional activity, thereby contributing to GC resistance, holds promise for improving GC sensitivity, potentially allowing for lower therapeutic doses [179]. Nevertheless, since such interventions risk activating alternative inflammatory cascades, further research is required to develop clinically safe strategies that overcome resistance and facilitate successful GC withdrawal.

### 8.2 Targeted glucocorticoid delivery

Novel biochemical strategies target GCs to specific tissues. A glucagon-like peptide-1 (GLP-1)-DEX co-agonist selectively delivers DEX to GLP-1 receptor-positive cells, enhancing glucose tolerance and insulin sensitivity while minimizing systemic exposure and side effects [180]. This targeted approach reduces systemic adverse effects and holds substantial potential for safer therapeutic applications in metabolic medicine.

### 8.3 11β-HSD1 inhibition

Since 11β-HSD1 regulates local GC bioavailability, its inhibition represents a promising strategy to reduce GC-related side effects. Elevated 11β-HSD1 expression appears in obese animals, metabolic disorder models, and liver-specific 11β-HSD1-overexpression transgenic mice. Tissue-specific 11β-HSD1 inhibition may mitigate GC-induced metabolic abnormalities by lowering local GC levels, as supported by evidence that liver-specific 11β-HSD1 overexpression exacerbates metabolic complications, including hepatic steatosis, dyslipidemia, de novo lipogenesis, and hypertension [148]. Conversely, whole-body *11β-HSD1* knockout mice are protected from metabolic dysfunction, remaining resistant to visceral obesity, insulin resistance, dyslipidemia, and hypertension, with enhanced lipolysis and favorable lipid profiles even under prolonged high-fat feeding [148], [181]. A preclinical study demonstrated that 11β-HSD1 inhibition ameliorates glucose handling and reduces obesity [181]. These findings collectively establish 11β-HSD1 as a promising therapeutic target for metabolic disease prevention.

Translational research has advanced toward clinical application through the development of both natural and synthetic 11β-HSD1 inhibitors. Multiple inhibitory approaches have shown efficacy across diverse metabolic contexts, H8 attenuates GC-induced hepatic lipid accumulation and injury through AMPK/SIRT1 pathway activation [182], while selective inhibitors such as AZD4017 have demonstrated significant efficacy in ameliorating GC-associated metabolic dysregulation, including insulin resistance, dyslipidemia, and hepatic steatosis [183]. These diverse findings support the development of 11β-HSD1 inhibitors as a multi-system therapeutic strategy. However, no 11β-HSD1 inhibitor has gained regulatory approval due to unresolved challenges such as limited tissue specificity, GC signaling complexity, and compensatory HPA axis activation after enzyme blockade.

### 8.4 Selective glucocorticoid receptor modulators

GCs reduce inflammation by suppressing activator protein-1 (AP-1) and NF-κB, yet produce metabolic side effects (obesity, T2DM, dyslipidemia, hypertension) through gene transactivation. Selective GR modulators (SEGRMs) represent a rational therapeutic strategy designed to preserve desired anti-inflammatory efficacy while minimizing the risk of adverse effects associated with conventional GC therapy.

SEGRMs achieve this dissociation by favoring GR monomer formation over dimerization, thereby blocking GRE-driven transactivation while maintaining transrepression-mediated anti-inflammatory effects [184]. Compound A (CpdA), a prototypical SEGRM, neither promotes GR dimerization nor GRE binding, thus blocking GR-mediated transactivation, while simultaneously exerting anti-inflammatory effects via upregulating NFE2L2 transcription [185]. In rodent models, B53, an SEGRM, efficiently relieves inflammation without transactivation activity [186]. Furthermore, CA-e, another SEGRM, demonstrates significant efficacy in mitigating GC therapy adverse effects [187]. Despite these promising preclinical results, no SEGRM has yet reached clinical application, highlighting the continued need for drug development and clinical validation to translate this therapeutic concept into clinical practice.

### 8.5 Alternative therapeutic strategies

Additional strategies target downstream proteins affected by GC treatment, narrowing the scope of GC effects and limiting side effects. Fibroblast growth factor 21 attenuates GC-induced oxidative stress, insulin resistance, dyslipidemia, and hepatic steatosis [188]. Metformin improves metabolic complications and clinical outcomes in patients receiving GC therapy [189]. Notably, weekly intermittent administration of synthetic GCs, rather than daily or continuous dosing, significantly improved skeletal muscle specific force in both male and female mice in a randomized preclinical study, suggesting novel GC-based therapy directions [190]. Finally, GC-induced metabolic side effects demonstrate substantial individual specificity, influenced by single-nucleotide polymorphisms and genetic variants affecting chromatin accessibility and three-dimensional chromatin architecture, highlighting the need for personalized approaches in GC therapy to optimize efficacy while minimizing metabolic complications [191]. Further research is essential to identify novel approaches to mitigate GC-induced metabolic complications.

## 9 Future perspective

Despite significant advances in understanding GC biology, several critical challenges remain unresolved. GC action mechanisms remain incompletely characterized, particularly regarding non-genomic GC effects, the functional balance between GR monomers and dimers, and tissue-specific GR signaling, all of which warrant deeper investigation. Novel strategies must urgently be developed to overcome GC resistance through targeted therapies addressing specific GC signaling pathways. Furthermore, identifying and targeting downstream proteins of GR activation may provide therapeutic options enhancing anti-inflammatory and immunosuppressive efficacy, including improved treatments for GC-induced osteoporosis arising from GR transrepression. The clinical translation of 11β-HSD1 inhibitors and SEGRMs should be prioritized, as these agents hold potential for reducing metabolic complications associated with both endogenous GC excess and prolonged high-dose GC therapy, though neither has yet gained regulatory approval. In clinical practice, individual variability in GR sensitivity necessitates personalized medication regimens, careful monitoring of drug-drug interactions, and dynamic dosage adjustment based on disease state. Future research must therefore advance our mechanistic understanding of GC action and enable the development of precisely regulated, next-generation GC therapies that maximize therapeutic benefit while minimizing adverse metabolic effects.

## 10 Conclusions

GCs exert diverse physiological effects through GR signaling, playing essential roles in metabolic homeostasis, immune regulation, and stress response. However, elevated GC levels or prolonged exogenous GC therapy cause significant metabolic complications, primarily through transactivation-mediated dysregulation of glucose and lipid metabolism, as well as HPA axis suppression. Current therapeutic strategies, including dose optimization, 11β-HSD1 inhibition, and selective GR modulators (SEGRMs), have shown promise in mitigating these adverse effects, yet none have achieved full clinical approval. Optimal GC management, therefore, requires individualized patient assessment and evidence-based strategies that carefully balance therapeutic benefit against metabolic risk.

## Abbreviations

α2-AR: α2-adrenergic receptor

11β-HSD1: 11β-hydroxysteroid dehydrogenase type 1

11β-HSD2: 11β-hydroxysteroid dehydrogenase type 2

ACE2: Angiotensin-converting enzyme 2

ACTH: Adrenocorticotropic hormone

AP-1: Activator protein-1

BAT: Brown adipose tissue

CBG: Corticosteroid-binding globulin

CpdA: Compound A

CRH: Corticotropin-releasing hormone

DBD: DNA-binding domain

ERK: Extracellular signal-regulated kinase

FTO: Fat mass and obesity-associated gene

GCs: Glucocorticoids

GLP-1: Glucagon-like peptide-1

GLUT: Glucose transporter

*GR*^*⁻/⁻*^: *GR* knockout

GR^dim^: GR dimerization-deficient

GREs: Glucocorticoid response elements

GRs: Glucocorticoid receptors

HDAC2: Histone deacetylase 2

HPA: Hypothalamic-pituitary-adrenal

HPG: Hypothalamic-pituitary-gonadal

HPT: Hypothalamic-pituitary-thyroid

IL: Interleukin

IRS: Insulin receptor substrates

JNK: c-Jun N-terminal kinase

Klf9: Krüppel-like factor 9

LBD: Ligand-binding domain

MAPK: Mitogen-activated protein kinase

MASLD: Metabolic dysfunction-associated steatotic liver disease

MC2R: Melanocortin-2 receptors

MetS: Metabolic syndrome

MRs: Mineralocorticoid receptors

nGREs: Negative glucocorticoid response elements

NO: Nitric oxide

NTD: N-terminal transactivation domain

PAI: Primary adrenal insufficiency

PDX1: Pancreatic-duodenal homeobox 1

PEPCK: Phosphoenolpyruvate carboxykinase

PI3K: Phosphatidylinositol 3-kinase

SEGRMs: Selective glucocorticoid receptor modulators

SGK1: Serum- and glucocorticoid-inducible kinase 1

T2DM: Type 2 diabetes mellitus

TA: Transactivation

TNF-α: Tumor necrosis factor-α

TR: Transrepression

VAT: Visceral adipose tissue

VLDL: Very low-density lipoprotein

## Ethics approval and consent to participate

Not applicable.

## Funding

This work was supported by the National Natural Science Foundation of China (82025008, 82525011, and 82171685).

## Data Availability

Not applicable.
